# Wrinkling Non-Spherical Particles and Its Application in Cell Attachment Promotion

**DOI:** 10.1038/srep30463

**Published:** 2016-07-27

**Authors:** Minggan Li, Dehi Joung, Bethany Hughes, Stephen D. Waldman, Janusz A. Kozinski, Dae Kun Hwang

**Affiliations:** 1 Department of Chemical Engineering, Ryerson University, 350 Victoria Street, Toronto, Ontario, M5B 2K3, Canada; 2 Li Ka Shing Knowledge Institute, St. Michael’s Hospital, 30 Bond Street, Toronto, Ontario, M5B 1W8, Canada; 3 Lassonde School of Engineering, York University, Keele Street, Toronto, Ontario, M3J 1P3, Canada

## Abstract

Surface wrinkled particles are ubiquitous in nature and present in different sizes and shapes, such as plant pollens and peppercorn seeds. These natural wrinkles provide the particles with advanced functions to survive and thrive in nature. In this work, by combining flow lithography and plasma treatment, we have developed a simple method that can rapidly create wrinkled non-spherical particles, mimicking the surface textures in nature. Due to the oxygen inhibition in flow lithography, the non-spherical particles synthesized in a microfluidic channel are covered by a partially cured polymer (PCP) layer. When exposed to plasma treatment, this PCP layer rapidly buckles, forming surface-wrinkled particles. We designed and fabricated various particles with desired shapes and sizes. The surfaces of these shapes were tuned to created wrinkle morphologies by controlling UV exposure time and the washing process. We further demonstrated that wrinkles on the particles significantly promoted cell attachment without any chemical modification, potentially providing a new route for cell attachment for various biomedical applications.

Particles with wrinkled surfaces are widely found in the nature, such as plant pollens, plant seeds (i.e. peppercorn and walnut) and microorganisms (i.e. neutrophils^1^), and they form various wrinkle morphologies and present in different sizes and shapes. These wrinkles with their significantly enlarged surface areas provide enhanced survival tools for natural particles, through modulation of pollen adhesion and hydration^2^, and regulating cell signalling^1^,^3^. Inspired by nature, synthetic particles with wrinkles exhibit extraordinary mechanical, morphological and photonic properties and have been used in various applications^4^,^5^,^6^,^7^,^8^. For example, the increased surface area greatly improves the performance in controlled drug delivery^9^,^10^, absorbent materials^11^, and inkjet recording^12^. The wrinkle morphology enhances the aerodynamics of aerosol in dry powder inhalation^10^,^13^,^14^ and optimizes aerodynamic drag control of bluff bodies^15^. Also, wrinkle alignment allows for self-assembly of microsystems^16^,^17^ and wrinkle geometrical patterns enable novel item tracking and identification technology^18^.

Artificially wrinkled particles are commonly formed in two separate steps: polymeric particles are first synthesized and then exposed to wrinkling post-processes to induce mismatched deformation between the particle cores and their skin layers^19^. In this wrinkling post-process, when particles are subjected to certain physical or chemical processing, such as particle drying^20^,^21^, surface chemical modification^22^,^23^, or additional material coating^24^, particle skin layers experience larger deformation than that of the particle cores, creating in-plane compressive stresses in the skin layers. When these compressive stresses exceed critical values, the skin layers buckle on the compliant particle cores, forming wrinkles on the particles.

In the majority of wrinkled particles created, the particles are commonly synthesized based on spray or emulsion methods followed by wrinkling post-processes. Although these particle-synthesis methods have been successfully used and their wrinkle morphology have been studied^19^,^25^,^26^, the particles made by these methods are limited to spherical or spheroidal shapes. In addition, due to batch process, these methods have difficulty in achieving size uniformity of the particles, which is critical in many applications such as controlled drug delivery and colloidal stability. Furthermore, lack of geometry constraints as a result of the shape limit mean guiding spatial patterns of wrinkling on spherical particles are challenging.

To better control particle size, shape and wrinkle morphology, which has been increasingly proven to be important in biomaterials design^27^, recent microfluidics-based methods have been adapted for wrinkled particle fabrication. In droplet-based microfluidic methods, although a narrow size distribution of particles can be achieved by microfluidic droplet formation, particles are still limited to spheroidal shapes^28^,^29^. As an improvement, mask-less lithography is applied to generate particles with desired size and shape, and enables geometry guided wrinkle morphology^18^. However, this wrinkling post-process is relatively lengthy, and ranges from 100–200 minutes, because it requires a chemical reaction for coating an additional layer of stiff material on the surface of the resulting particles^18^. Further, this uniform coating makes it difficult to generate complex wrinkle morphologies, such as hierarchy wrinkles, on the particles, which requires multiple layers of materials with gradient physical properties in the skin to form different wavelengths of wrinkles^30^.

Here, we introduce stop flow lithography (SFL) in combination with a rapid wrinkling post-process (in the range of a few seconds) to generate wrinkled non-spherical particles, not only with designed particle size and shape, but also with hierarchy and tuneable wrinkle morphology. These artificially wrinkled particles show unique surface functions and significantly promote cell attachment to the particles in cell culture studies, without any additional chemical treatments.

## Results and Discussions

### Wrinkled particle fabrication

**Figure 1:**
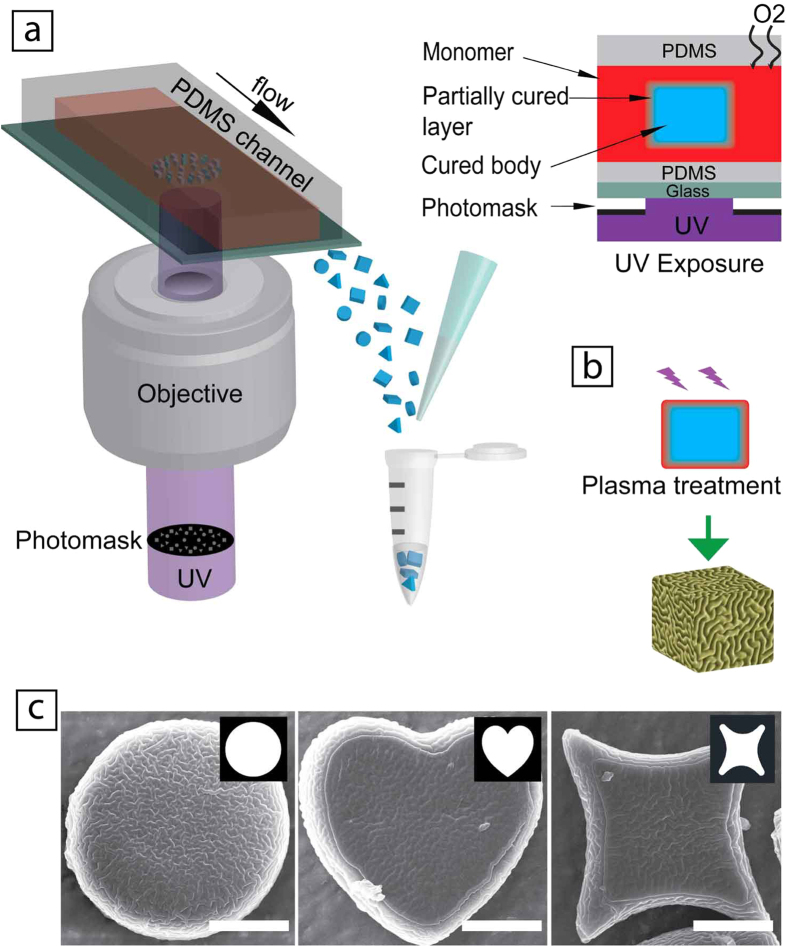
A microfluidic-based method for wrinkled particles fabrication. (**a**) Schematic of the particle generation using a stop-flow lithography technique and the mechanism of the particle polymerization and PCP layer formation by oxygen inhibition. (**b**) After washing away the uncured monomers on the particle surface, the PCP layer turned into wrinkles when plasma treated. (**c**) Wrinkled particles with designed shapes obtained from this method. Scale bars are 20 um.

In this method, for the particle fabrication by using SFL technique, a photo-curable monomer flow is stopped in a microfluidic channel and subsequently a UV beam through a photomask is exposed to the channel to polymerize the monomer into photomask-defined shapes. Immediately after the particle formation, the monomer flow is resumed and the synthesized particles are discharged from the channel (Fig. 1a) and washed as part of the post-wrinkling process. This process is repeated and particles are generated in a continuous and high throughput fashion.

During the UV-induced polymerization, oxygen diffusion into the channel through the PDMS walls plays critical roles in the particle discharge and wrinkle formation as well. As UV is exposed to the channel, polymerization starts from the middle of the channel height, where oxygen is depleted first by the UV initiated free radicals. The polymerized particle rapidly grows as the UV exposure continues. As the particle reaches the channel walls, its growth slows due to continuous oxygen diffusion from the walls. As the UV beam is turned off, the polymerization stops and particles are formed with a polymerization gradient. Thus, by controlling UV expose time, one can create not only uncured monomer layers (lubrication layers) at the channel walls to ensure the smooth discharge of the formed particles, but also particles with fully cured body and partially cured polymer (PCP) outer layers (Fig. 1a)^31^,^32^. This PCP outer layer will also serve as the skin layer, later in the post-wrinkling process.

The PCP outer layer of a particle is comprised of a loose PEG polymeric network and uncrosslinked monomers trapped inside the network. Since these trapped monomers are not chemically bond to the PCP network, they can be completely removed by a thorough wash or can be partially retained by a controlled wash using a washing agent that contains a known concentration of monomers—higher concentrations will leave more monomers trapped and lower concentrations will remain less monomers in the PCP layer. After a controlled wash particles are subjected to plasma treatment for the wrinkling post-process, the PCP skin layer condenses with the retained monomers into a crust and simultaneously expands the layer by providing crosslinking and annealing while having little effect on the particle core^7^. This mismatched deformation between the core and crust layer, induced by the plasma, buckles the crust, forming wrinkled particles (Fig. 1b); this wrinkling formation process is completed in only 5 seconds in our current setup.

For a given height of a PDMS channel, photomask features define the 2D extruded shapes of particles, while the mask feature-size and the magnification of microscope objective used determine particle sizes. Thus, by simply selecting photo-mask patterns and objectives, we can facilely produce particles with unlimited 2D extruded shapes, or even 3D shapes^32^, and desired sizes. Figure 1c shows wrinkled particles with selected shapes and sizes obtained from our method by using the corresponding masks. At a small size scale, the free radical diffusion during UV exposure may introduce undesired polymerization around a designed particle and affect the final particles size, a minimum feature size of 5 um can be obtained using our current system according to our previous study^31^.

### Effect of UV exposure time on wrinkle morphology

**Figure 2:**
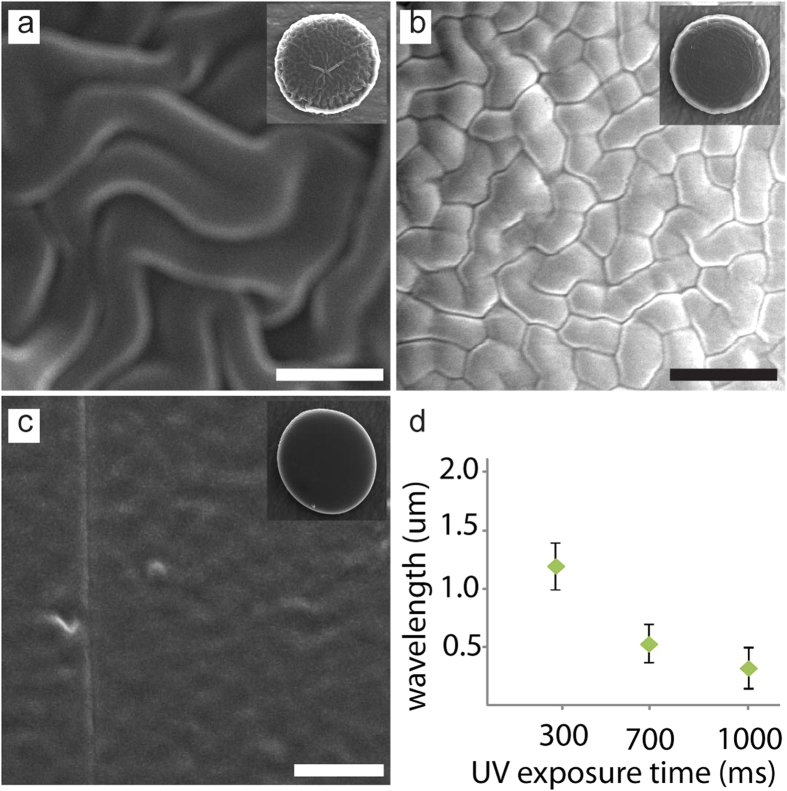
Wrinkle morphologies on particles tuned by UV exposure time with the same washing solutions (5% PEG-DA water solution). (**a**) 300 ms, (**b**) 700 ms and (**c**) 1000 ms. (**d**) The wavelengths of the wrinkles on particles produced with different UV exposure times. Each data points represent 10 wavelength measurements. Scale bars are 2 um.

During UV-polymerization for particle formation, oxygen inhibition causes a nonlinear profile of polymer conversion rate from the center to the outer layer of the resulting particles. Thus, by carefully tuning UV exposure time, we can control the PCP layer thickness of particles and their polymer conversion rate (see the simulation results of the UV polymerization process with oxygen inhibition in Figure S1), which in turn determines the wrinkle wavelength upon plasma treatment^31^. The wavelength of the wrinkles, *λ*, can be approximated by 
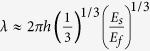
; where *h* is the thickness of the crust layer after plasma treatment, and *E*_*S*_ and *E*_*f*_ are the Young’s moduli of the crust layer and the particle core, respectively. In particle formation process, UV exposure time not only determines the ratio of (*E*_*S*_/*E*_*f*_)^7^, but also dominates the thickness (*h*) of the PCP and the crust layer after plasma treatment. Consequently, the UV exposure time enables us to control the wavelength of the wrinkles. Figure 2 shows the morphology of wrinkled particles and their corresponding wrinkle wavelengths by using the UV exposure time of 300, 700 and 1000 ms, after washing with a 5% PEG-DA solution and plasma treatment for 5 sec. As the UV exposure time increases from 300 ms to 1000 ms, the wavelength decreases from a micron scale of 1.3 μm to submicron of 0.4 μm (Fig. 1d). Along with the decrease of the wavelength, the amplitude of the wrinkles also decreases. The morphology change can be also observed which varies from a continuous to discontinuous pattern as the UV exposure time increases from 300 ms to 1000 ms, as shown in Fig. 2a–c.

### Effect of monomer concentration of washing agent on wrinkle morphology

**Figure 3:**
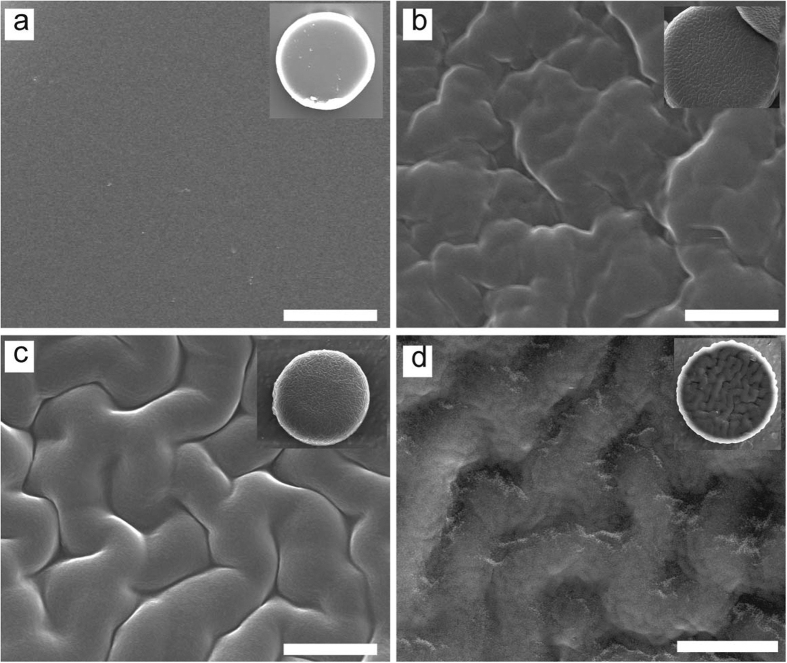
Wrinkle morphologies on particles tuned by washing agent using the same UV exposure time. After particle synthesis, the particles are washed by a solution with different concentrations of PEG-DA in water. (**a**) 0% PEG-DA solution wash removes all the monomers and thus produce smooth surface, (**b**) 5% PEG-DA solutions wash and (**c**) 10% PEG-DA solutions wash leave different levels of monomers and make different wrinkle patterns. (**d**) 25% PEG-DA solutions wash leaves excessive monomers on the surface and lead to nested wrinkles. Scale bars are 2 um (**a–c**) and 10 um (**d**).

In addition to the UV exposure time, we can also control the morphology of wrinkles on particles by tuning the concentration of monomers used in the washing agent. By using various concentrations of solutions for wash, different amounts of monomers can be retained in the PCP layer and thus result in different morphologies of wrinkles after plasma treatment. For this experiment, we fixed the UV exposure time at 200 ms and washed the particles with 0%, 5%, 10% and 25% of PEGDA solutions. As a result, these particles display different morphologies after plasma treatment, as seen in Fig. 3. When washed by 0% solution, the monomers in the PCP layer were completely removed, thus no wrinkles were formed and smooth surface was observed (Fig. 3a). After increasing the monomer concentration in the washing solutions, the wavelength of wrinkles increased and various patterns were observed. When washed with a high concentration monomer solution (25%), excessive monomers were retained in the PCP layer and hierarchy (nested) wrinkle morphology presented. The plasma treatment initially generates primary wrinkles on the surface and its amplitude quickly saturates; this saturated surface experience further compressive stresses in the primary wrinkled crust and generate secondary wrinkles with a larger wavelength, forming a hierarchy (nested) wrinkle patterns on the surface^4^ (Fig. 3d).

### Cell attachment to wrinkled particles

**Figure 4:**
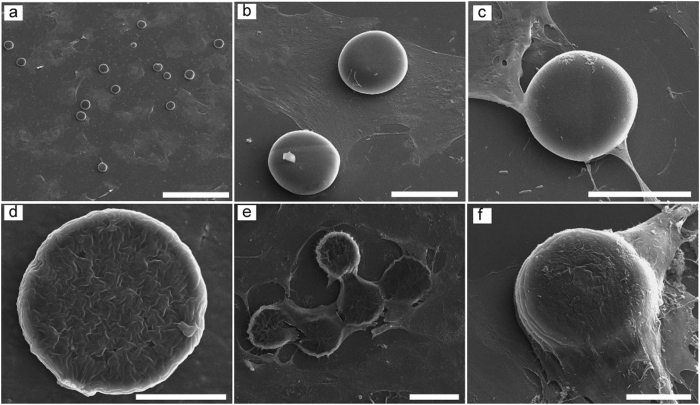
Cell experiments show cells ignore smooth particles but prefer to attach to wrinkled ones. (**a,b**) SEM images show cells spread on substrate and ignore the smooth particle. (**c**) A representative SEM image shows that although some cells attach to smooth particles, they only anchored to the particle edges. (**d**) A representative SEM image shows the surface morphology of the wrinkled particles before cell seeding. (**e**) Cells attach to wrinkled particles after 3 days cell culture. (**f**) When cells attach to wrinkled particles, they climb onto the particles and conform to them. Scale bars are 300 um (**a**), 40 um (**b,c,e**), 20 um (**d,f**).

The unique surface morphology of wrinkled particles produced by our method may find various applications. In particular, we demonstrate that a simple physical modification on particle surface, without any intensive chemical modification, can greatly enhance cell attachment. In this experiment, we used smooth and wrinkled particles of 40 μm in diameter – to create the particles by using a UV exposure time of 300 ms but with two different concentrations of washing solutions: 0% and 10% monomer solutions for the smooth and wrinkled particles, respectively. We randomly seeded bovine fibroblasts onto both the smooth and wrinkled particles. After a three day cell culture, in the case of the smooth particles, cells spread onto the substrate, generally ignoring the particles (Fig. 4a,b). Although some cell attachment to the smooth particles are observed, these cells only reach to the edge of the particles by a small partial anchorage (Fig. 4c). However, in case of the wrinkled particle samples (a representative surface morphology of the wrinkled particles before cell seeding is shown in Fig. 4d), cells attached to and covered most of the wrinkled particles (Fig. 4e). With the samples of 300 wrinkled and smooth particles, about 75.1% (±6.9%) of the wrinkled particles were attached by cells, whereas cells attached to only 17.6% (±8.5%) of the smooth particles (Figure S3).

**Figure 5:**
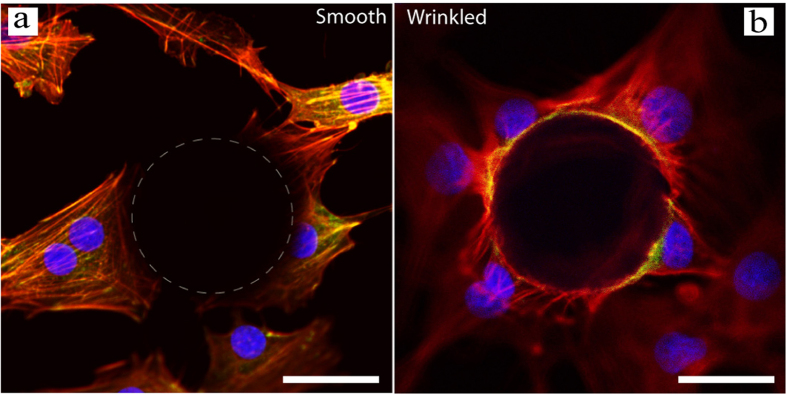
Confocal microscope study of cell attachment on smooth and wrinkled particles. Fluorescence images of cells around smooth (**a**) and wrinkled (**b**) particles by confocal microscopy. The actin networks, focal adhesions (co-localization of actin and vinculin) and cell nuclei are shown in red, yellow and blue, respectively. The dashed circle in the left hand image (**a**) indicates the smooth particle. Scale bar are 30 um.

In addition, when cells attach to wrinkled particles, the cells climbed and conformed onto the particles, occupying a large surface area of the particle (Fig. 4f), which was not observed on the smooth particles. This is also confirmed by a confocal microscopy study. For the smooth particle, the cells appeared to ignore the smooth particles and attached only to the substrate, indicated by actin networks appearing only on the substrate. There were no focal adhesions on the particle surface (Fig. 5a). In contrast, on the wrinkled particle, cell attachment to the surface of the particles was observed with actin networks appearing to extend predominantly outwards from the top edge of the particle and focal adhesions at the particle edges (Fig. 5b).

**Figure 6:**
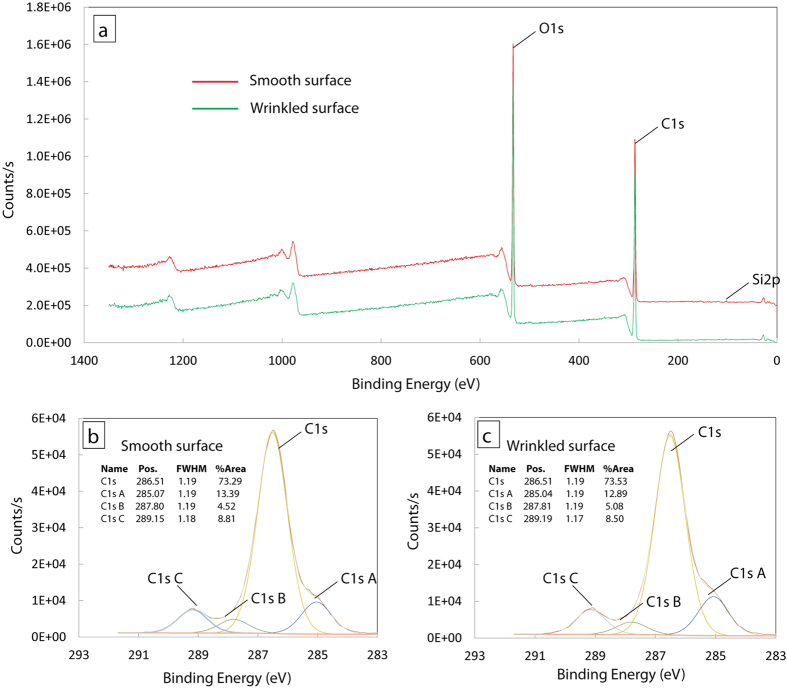
XPS analysis of smooth and wrinkled samples. (**a**) XPS survey of smooth and wrinkled samples. The plot for smooth sample is 2.0 × 10^5^ shifted for comparison. (**b,c**) are the high resolution scans of C1s for smooth and wrinkled surfaces.

XPS analysis was carried out to examine the surface chemistry of smooth and wrinkled particles. Both wide scans and high resolution scans show that no significant chemical difference between the surfaces of smooth and wrinkled particles (Fig. 6). Thus the cell attachment to wrinkled particles can be attributed to the surface topology of wrinkles. This further suggest that the wrinkles alone can improve cell attachment, which usually achieved by complex chemistry modifications^33^,^34^. Our findings provide a simple route to promote cell attachment to particles by its wrinkled surface textures without chemical processing, which will be beneficial to a number of biomedical applications where particles are used as cell microcarriers such as cell delivery in cellular therapy^35^,^36^, tissue formation and regeneration in tissue engineering^37^,^38^, and cellular biophysical studies^39^.

In addition to the cell attachment, these wrinkled particles may provide useful functions in other biomedical applications. In drug delivery, wrinkled particles can increase drug carrying efficiency with enhanced surface area and reduce inter-particulate adhesion by the roughness of wrinkles, thus significantly improve drug delivery performance^9^,^40^. Furthermore, the size- and shape-controllable ability of our method may allow the particles to be designed to achieve optimal drug delivery performance^41^,^42^.

## Conclusion

In summary, by taking advantage of partial polymerization in SFL in combination with plasma treatment, we have developed a simple and rapid route for wrinkled particle fabrication. The effects of UV exposure time and monomer concentration of washing agent on the wrinkle morphologies were studied and the results indicated that this method can not only fabricate wrinkled particles with designed shape and size, and also create wrinkles on particle surfaces with tuned morphology and wavelength. The wrinkling post-process is completed in a few seconds with plasma treatment, thus greatly simplifying the wrinkled particle formation. Furthermore, we demonstrated that the surface wrinkles significantly improved cell attachment to the particles without any chemical modifications in our cell culture study. These findings may be beneficial to many biomedical applications where cell attachment to a surface or particle is desired, such as cell micro-carriers, cell physiological study and tissue engineering.

## Methods

### Materials

Poly(ethylene glycol) (700) diacrylate (PEG-DA 700, Sigma-Aldrich) and 2- hydroxy-2-methylpropiophenone (Darocur 1173, Sigma-Aldrich) initiator are used for polymeric particles synthesis. 5% Darocur 1173 in PEG-DA 700 were used as the prepolymer solutions for all the particles synthesis.

### Microfluidic Device Fabrication

A mixture of polydimethyl-siloxane elastomer (PDMS, Sylard 184, Dow Corning) and curing agent at ratio of 10:1 were prepared to make the microfluidic channels. The elastomer mixture was poured onto a SU-8 patterned silicon wafer (SU-8 photoresist, Microchem) and baked in an oven at 65 °C for 1 hour in order to mold the PDMS channels. The channels were then placed onto PDMS-coated glass slides, where the PDMS layer is partially cured at 65 °C for 20 minutes. The assembled channels were then baked for another 1 hour for full cure of the PDMS channels and the coated layers.

### Photopolymerization Setup

Microparticles were polymerized by using stop-flow lithography and designed photomasks. A metal arc lamp (Lumen 200, Prior Scientific, Rockland, MA, USA) was connected to the Axio Observer inverted microscope to provide the UV source, and a UV shutter (Lambda SC, Sutter Instruments, Novato, CA, USA) was used to control the UV exposure. The prepolymer solution was supplied through a pneumatic tubing system, which consisted of a pressure regulator (Type 100LR, ControlAir, Amherst, NH USA), serially connected to a three-way solenoid valve (Model 6014, Burkert, Germany) and PDMS channel. The UV shutter and the solenoid valve were controlled by a program in Labview (National Instruments, Austin, TX, USA) through a digital controller (NI 9472, National Instruments, Austin, TX, USA) to control UV exposure time and prepolymer flow cycle. The microscope equipped with 5 x /0.13, 10 x /0.3, and 20 x /0.4 objectives (N-Achroplan, Ec plan-Neofluar and korr LD Plan-Neofluar, Carl Zeiss, Jena, Germany) was used as the synthesis platform. The desired UV excitation (350 nm) required by polymerization was attained by filtering the UV light source through a UV filter set (11000v3, Chroma, VT, USA). The UV intensity is 280 mw/cm^2^ after passing the filter set. AUTOCAD 2011 was used to design the transparency photomasks. Photomasks were printed at a resolution of 25 000dpi (CAD/Art Services, OR, USA).

### Polymeric Particle Synthesis

Monomer solution consisting of 95% PEG-DA 700 and 5% Darocur 1173 was supplied to the channel. When flow was fully stopped, UV exposure at different times through the designed photomasks and a 10X objective lens were applied to polymerize desired non-spherical particles. Particles remain in the prepolymer solution and are collected at the outlet. The solution is diluted with DI water at different ratios to partially or completely remove the uncrosslinked monomers. After the dilution process, samples were loaded on glass slides and dried for plasma treatment.

### Wrinkle formation

Before plasma treatment, the dried particle samples were transferred onto aluminum foil to ensure the plasma contact of the bottom of the particles. See the surface morphology of the aluminum foil in Supplementary Information (Figure S2). All samples were exposed to plasma for 5 seconds (Harrick Plasma, PDC-32G, NY).

### Cell culture and imaging

Bovine ligament fibroblasts were randomly seeded onto the wrinkled and smooth particle samples at a cell density of 3 × 10^4^ cells/cm^3^. After 72 hours of culture, samples were fixed and dried for SEM imaging (see supplementary information for cell culture and Immunofluorescence staining).

### Simulations

Simulations were carried out by solving the mass transport equations for oxygen and monomer in the channel using Comsol Multiphysics 4.3b (Comsol, Inc.). Details can be found in Supplementary Information.

### SEM

Samples on aluminum foil were transferred onto SEM mounts, sputtered with 5 nm platinum and observed by a field emission scanning electron microscopy (Quanta 3D FEG SEM, FEI Co., OR) at HV 20.00 kV with a ETD detector.

### XPS analysis

After plasma treatment, both smooth and wrinkled samples were soaked in DI water for 24 hours to remove any chemical residuals in the samples. The XPS survey was scanned with Constant Analyser Energy (CAE) 200.0 and at resolution of 1.00 eV. The high resolution scans for C1s was performed at 0.10 eV with CAE 25.0.

## Additional Information

**How to cite this article**: Li, M. *et al*. Wrinkling Non-Spherical Particles and Its Application in Cell Attachment Promotion. *Sci. Rep*. **6**, 30463; doi: 10.1038/srep30463 (2016).

## Supplementary Material

Supplementary Information
