# Supplementary material for: Wrinkling Non-Spherical Particles and Its Application in Cell Attachment Promotion
Source: Sci Rep. 2016 Jul 27;6:30463. doi: 10.1038/srep30463 (PMC4962049; doi:10.1038/srep30463)
Supplement: Supplementary Information [file srep30463-s1.pdf]

Supplementary Information

Wrinkling Non-Spherical Particles and Its Application in Cell Attachment Promotion

Minggan Li[1,2], Dehi Joung[1], Bethany Hughues[1], Stephen D. Waldman[1,2], Janusz A. Kozinski[3], and Dae Kun Hwang[1,2*]

[1]Department of Chemical Engineering, Ryerson University
350 Victoria Street, Toronto, Ontario, M5B 2K3, Canada

[2]Li Ka Shing Knowledge Institute, St. Michael's Hospital,
30 Bond Street, Toronto, Ontario, M5B 1W8, Canada

[3]Lassonde School of Engineering, York University,
4700 Keele Street, Toronto, Ontario, M3J 1P3, Canada

Correspondence to Prof. D. Hwang* E-mail: dkhwang@ryerson.ca

1. Simulation of UV polymerization in PDMS channel. As UV is shined to a PDMS channel filled with a prepolymer solution, free radicals released from photoinitiator crosslink the PEGDA monomers while oxygen in the solution inhibit the polymerization. The expressions for the specie change in time and space can be obtained from the mass transport equations for oxygen and monomer, respectively. To simulate the polymerization process during UV exposure, we solve these equations in Comsol 4.3b. The dimensionless oxygen concentration (σ) is[1]:

$$\frac{\partial\sigma}{\partial\tau} = \frac{\partial^2\sigma}{\partial\zeta^2} + \frac{H^2}{\omega_0{}^2}\frac{\partial^2\sigma}{\partial\rho^2} - Da_1\sigma\left(-\sigma + \sqrt{\sigma^2 + \alpha I'\exp(-\beta\zeta)}\right), \tag{1}$$

where,

$$\tau = {}^{tD_O}\!/_{H^2}\,,\ \sigma = {}^{[O_2]}\!/_{[O_{2,eqb}]}\,,\ \zeta = {}^{z}\!/_{H}\,,\ \rho = {}^{r}\!/_{H}\,,$$

$$Da_1 = \frac{k_O^2 H^2 [O_{2,eqb}]}{2k_t D_O},\ \alpha = \frac{4\varphi\varepsilon_1[PI]I_0 k_t}{k_O^2[O_{2,eqb}]^2},\ \beta = \varepsilon[PI]\,H.$$

H is the channel height, $[O_{2,eqb}]$ is the equilibrium concentration of oxygen in the precursor solution, k_t is the termination reaction constant, φ is the quantum yield of formation of free-radicals, and [PI] is photoinitiator concentrations. I_0 is the intensity of incoming light, ε is the extinction coefficient of photoinitiator. $[O_2]$ is the concentration of oxygen, D_O the diffusivity of oxygen in the precursor solution, and k_O the oxidation reaction constant.

The dimensionless monomer concentration (ξ) is[1]:

$$-\frac{\partial\xi}{\partial\tau} = D_{a_2}\xi(-\sigma + \sqrt{\sigma^2 + \alpha\, I'\exp(-\beta\zeta)}), \tag{2}$$

where k_p is the polymerization reaction constant and $[M]$ is the concentration of monomer in the precursor solution. $\xi = \frac{[M]}{[M_0]}$ with $[M_0]$ the monomer initial concentration. $D_{a_2} = \frac{k_O k_p H^2 [O_{2,eqb}]}{2k_t D_O}$.

The detailed derivation of the equations and the solutions of these equations can be found in our group's previous work[1], where most the parameter values can also be found, except the channel height, which is 60 μm in this work.

In the region that monomer concentration is lower than 0.98, there is more than 2% monomer conversion (1- ξ) at which point the monomer solution solidifies into a gel.[2] The region with less than 2 % monomer conversion remains liquid and can be rinsed easily. The simulations in Figure S1 show the UV polymerization progress of a half particle. The partially polymerized outlayer forms a loose polymer network that can trap uncured PEGDA monomers inside. With the increase of the UV exposure time, the cured particle grows while the partially cured polymer outlayer becomes thinner.

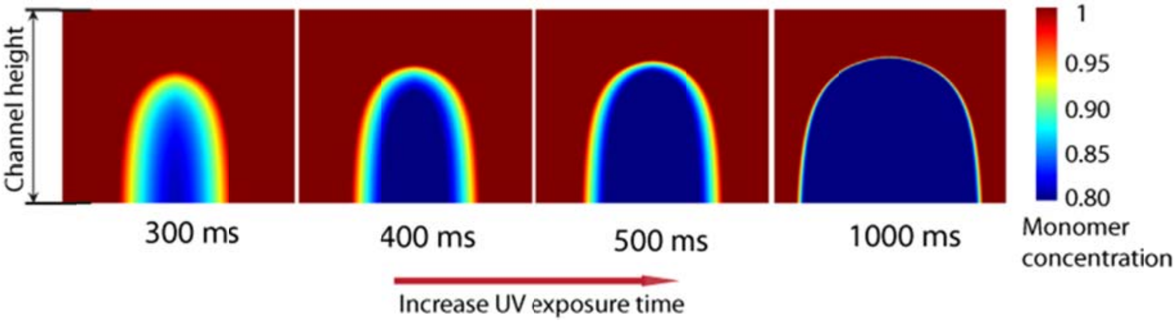

Figure S1| Simulation of the UV polymerization process with oxygen inhibition for a half particle. It shows the formation progress of a particle with the UV exposure time. As the increase of the UV exposure time, the size of the particle core increases while the partially cured polymer (PCP) layer thickness decreases.

2. Cell isolation. Bovine fibroblasts used in the cell experiments were isolated from the central ligament of the metacarpal-carpal joint of 12–18 month old calves. Harvested ligaments were cleaned of extraneous fat and cut into < 1 mm^3 pieces, and then digested in DMEM culture media containing 0.25% collagenase A (Roche Diagnostics Corporation) for 36 hours at 37ºC.

Viable cells were seeded in culture flasks and maintained in DMEM medium containing 5% FBS (Sigma-Aldrich Inc.) and antibiotics (100 U/mL penicillin, 100 mg/mL streptomycin) (Invitrogen). Cell cultures were grown in an incubator maintained at 37ºC and 95% relative humidity supplemented with 5% CO_2. Cells were cultured up to passage 5 for the experiments with media changes every 2-3 days.

3. Cell culture. Smooth and wrinkled particles were placed on glass slides separately and were soaked in deionized water for 2 days to remove uncured PEGDA monomers and unreacted photo initiator. The particle samples were then immersed in 70% ethanol for 30 minutes for sterilization, followed by washing with 1X PBS (pH 7.4). Slides were then placed in cell culture dishes. Cells were seeded onto the substrates at a density of 3×10^4 cells/cm^2 in a small volume of complete media (DMEM containing 5% FBS and antibiotics) and allowed to attach for 4 hours. Following cell attachment, a 1 mL of complete media was added to the cultures. After 72 hours of culture, samples were rinsed with PBS (pH 7.4), fixed with 4% paraformaldehyde (Bioshop) overnight at 4ºC and then dehydrated with graded ethanol solutions (70, 90, 95, and 100%). Cultures were then chemically dried by grading to 100% hexamethyldisilazane (33, 67, and 100%, Sigma-Aldrich Inc) followed by air-drying in a chemical hood overnight.

4. Fluorescence imaging. After 72 hours of cell culture, samples were rinsed by PBS 3 times followed by 30 min. incubation. They were then stained by live/dead cell viability assay agent (L-3224, Invitrogen, Canada) for 10 min. Both bright field and fluorescent images were taken by by a charge coupled device (CCD) camera (QImaging, Canada). Images were then merged together using ImageJ for analysis.

5. Immunofluorescence staining and imaging

To identify cellular focal adhesions, microfilament locations and the cellular nucleus, immunofluorescence staining was performed. To stain for F-actin, TRITC-conjugated Phalloidin (Part No. 90228) (15 μg) was reconstituted by suspending into 250 μL of methanol. For future use the TRITC-conjugated Phalloidin was aliquot into 50μL into tubes and stored at -20°C.

Cells were fixed with 4% paraformaldehyde in 1x PBS for 15-20 minutes at room temperature. Cells were then washed twice with 1xPBS with 10mM Glycine. Permeabilize cells with 0.1% Triton X-100 in 1x PBS for 1-5 minutes at room temperature. Washed twice with 1xPBS. Blocking solution of 3% bovine serum albumin (BSA) in 1xPBS was applied for 60 minutes at room temperature. Dilute the primary antibody (Vinculin Monoclonal Antibody, purified clone 7F9 (Millipore Cat. No. 90227)) to a working concentration of 1/500 in blocking solution of 1%BSA in PBS and incubate for 1 hour at room temperature. Wash three times (5-10 minutes each) with 1xPBS. Dilute secondary antibody to 1/500 (Gt x Ms, FITC-conjugated, Millipore Cat. No. AP124F) in 1x PBS just before use and incubate for 60 minutes at room temperature. Double labeling was performed during the secondary antibody stain by diluting TRITC conjugated Phalloidin to 1/1000. Wash was performed three times (5-10 minutes each) with 1x PBS. Following the washing step, nuclei counterstaining was performed by incubating cells with DAPI (Millipore Cat. No. 90229) at a dilution of 1/1000 for 5 minutes at room temperature, followed by washing cells three times (5-10 minutes each) with 1xPBS. Cells were mounted on a coverslip using antifade mounting solution (Vectashield mounting medium, H-1200) and visualized with a Confocal Zeiss LMS700 with ZEN Black acquisition software.

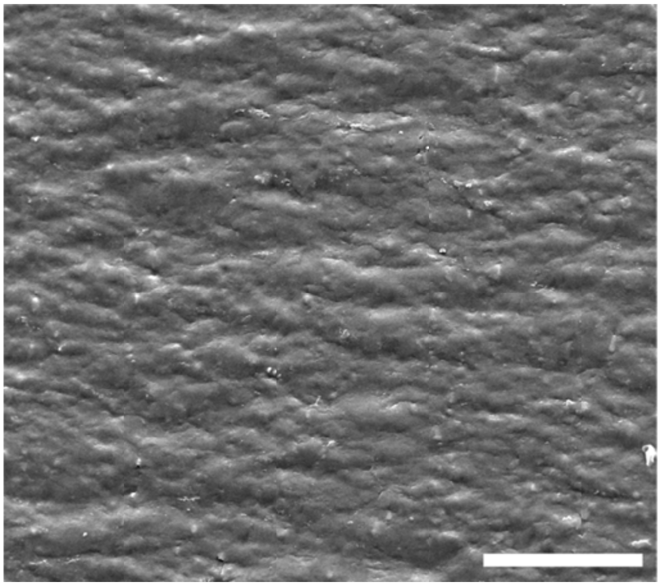

Figure S2| SEM image of the morphology of the aluminum foil surface. The rough surface may allow for the plasma treatment of the bottom sides of particles. Scale bar is 50 um.

Smooth particle samples Wrinkled particle samples

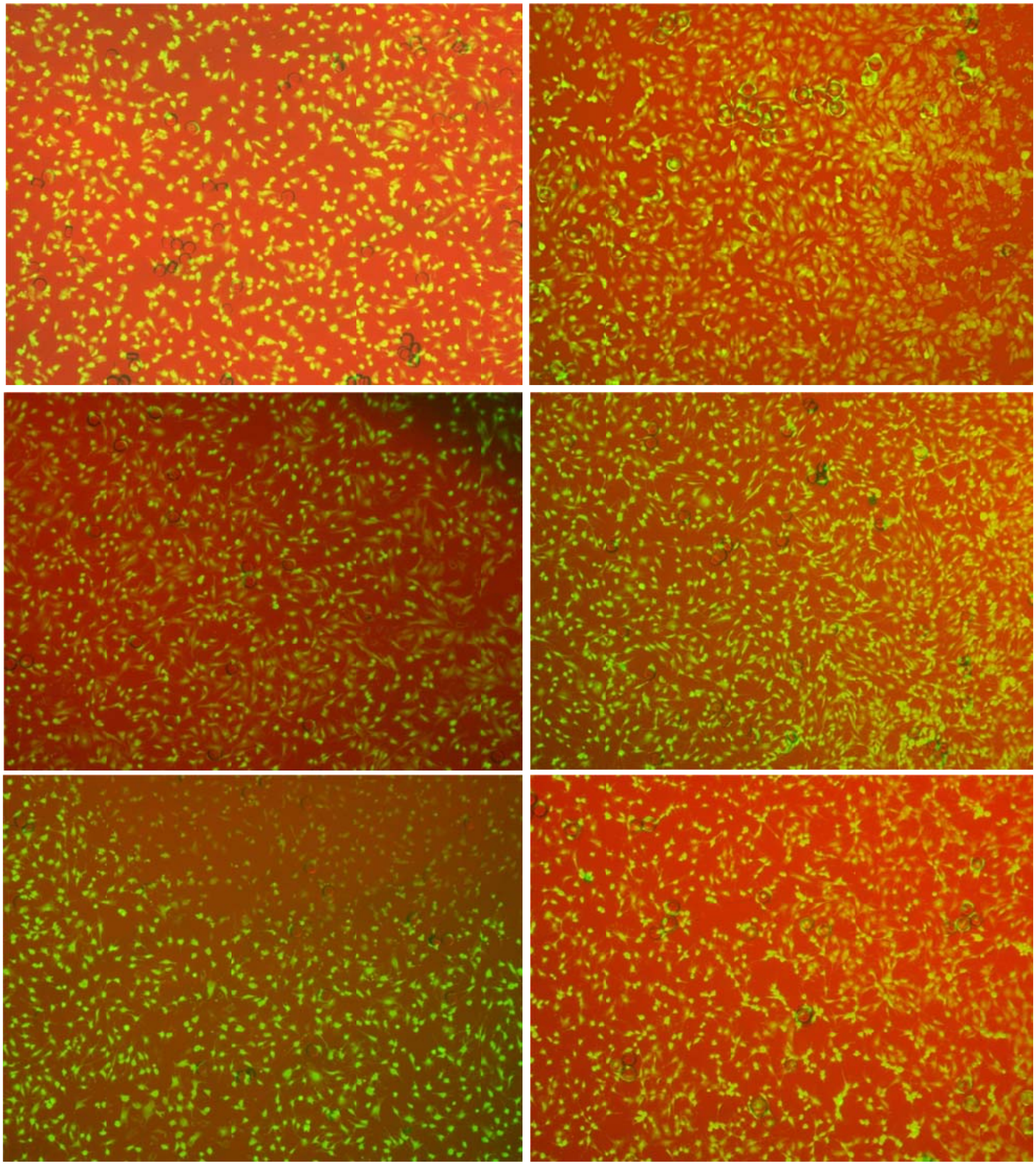

Figure S3|. Examples of cell cultures on smooth and wrinkled particles. Images for cells display fluorescence green and images for substrate and particles are phase-contrasted. Then two images were merged by using Image J.

Reference:

[1] N. Hakimi, S. S. Tsai, C. H. Cheng and D. K. Hwang, *Adv Mater*, 2014, 26, 1393-1398.

[2] Andrzejewska, E. *Prog. Polym. Sci.* **2001**, *26*, 605.
